# Acute effects of resistance exercise on skeletal muscle glycogen depletion: A systematic review and meta‐analysis

**DOI:** 10.14814/phy2.70683

**Published:** 2025-12-19

**Authors:** Amin Hamidvand, Slaheddine Delleli, Jeffrey A. Rothschild, Farzaneh Chenaghchi, Afshar Jafari, Alireza Naderi

**Affiliations:** ^1^ Department of Biological Sciences in Sport, Faculty of Sport Sciences and Health Shahid Beheshti University Tehran Iran; ^2^ High Institute of Sport and Physical Education University of Sfax Sfax Tunisia; ^3^ Physical Activity, Sport, and Health, UR18JS01 National Observatory of Sport Tunis Tunisia; ^4^ Sports Performance Research Institute New Zealand Auckland University of Technology Auckland New Zealand; ^5^ High Performance Sport New Zealand Auckland New Zealand; ^6^ Department of Exercise Physiology, Faculty of Sport Sciences University of Guilan Rasht Iran; ^7^ Department of Sport Physiology, Faculty of Human Sciences Borujerd Branch, Islamic Azad University Borujerd Iran

**Keywords:** Carbohydrate, energy, exercise, metabolism, performance

## Abstract

To compile and statistically summarize quantitative evidence on the acute effects of resistance training sessions on muscle glycogen concentration, a systematic search was conducted on Pubmed, Web of Science, and Scopus databases up to 28th July 2024. Twenty studies including 168 male and 12 female participants were eligible. A multilevel, random‐effects meta‐analysis was used to calculate the overall mean difference (MD) with a 95% confidence interval (CI) and prediction interval (PI). The model (28 effect sizes across 20 clusters) revealed a significant glycogen decrease (MD = −104.3; 95% CI: −137.6 to −71.0; PI: −244.4 to 35.7; *p* < 0.001). Meta‐regression showed greater depletion with more sets (Estimate = −11.2; 95% CI: −18.0 to −4.3; *p* = 0.001) and longer session duration (Estimate = −1.3; 95% CI: −2.3 to −0.3; *p* = 0.009), but less with higher intensity (Estimate = 2.88; 95% CI: 1.2 to 4.5; *p* = 0.0006). Subgroup analysis showed greater depletion with varied intensity (MD = −162.9) versus fixed (MD = −82.5), and in untrained (MD = −113.0) versus trained participants (MD = −101.3). A single resistance training session depletes glycogen in the vastus lateralis muscle, with depletion influenced by training intensity, session duration, number of sets within session and training status.

## 1 INTRODUCTION

Nearly all athletes perform resistance training to improve strength, power, muscle hypertrophy, and endurance capacity (Aagaard & Andersen, 2010; Hickson, 1980; Kraemer et al., 1995). During both resistance and endurance exercise, carbohydrate and fat serve as primary fuel sources, with dietary carbohydrate stored as glycogen in the liver and muscles playing a particularly prominent role during moderate‐ to high‐intensity activities, including resistance training (Hargreaves & Spriet, 2020; Vigh‐Larsen et al., 2021). A single bout of resistance exercise may deplete muscle glycogen by 24%–40% (MacDougall et al., 1999a; Tesch et al., 1986), depending on exercise intensity, duration, and the number of muscle motor units recruited (Iraki et al., 2019).

For athletes performing multiple training sessions within ~24 h, post‐exercise carbohydrate ingestion becomes increasingly important to support subsequent performance (Haff et al., 1999). Initiating exercise with low muscle glycogen levels accelerates fatigue, impairs isotonic force production and isometric strength (Jacobs et al., 1981; Knuiman et al., 2015), and can hinder subsequent exercise performance (Cholewa et al., 2019; Haff et al., 2003). This decline is attributed to disruptions in the excitation‐contraction (E‐C) coupling process, which involves altered cell excitability and Ca^2+^ kinetics (Ørtenblad et al., 2011). These effects may stem from a direct metabolic impact such as limited ATP production from localized energy metabolism or indirect mechanisms, including feedback regulation linked to depleted muscle glycogen and its effects on the central nervous system (Vigh‐Larsen et al., 2021). Research consistently highlights the importance of daily carbohydrate consumption, particularly pre‐ and post‐exercise, in optimizing glycogen stores and supporting exercise performance (Naderi et al., 2016, 2023).

Several studies have indicated that different exercise modalities trigger various levels of glycogen depletion including 60–90 min simulated soccer matches by 38%–53% (Gunnarsson et al., 2013), ice hockey games by 31%, (Thorsteinsson et al., 2023) and 1–15 min submaximal cycling exercise, which led to glycogen depletion from ~8.5% and 12%, to 30% and ~42% in subsarcolemmal and intramyofibrillar levels, respectively (Schytz et al., 2024). Although sports nutrition guidelines related to carbohydrate recommendations in endurance exercise have received considerable attention (Naderi et al., 2023), recommendations for strength‐trained athletes and bodybuilders remain less considered. Several publications suggested carbohydrate ingestion in ranged doses from 3 to 10 g/kg per day for those who engage in resistance/power exercises and bodybuilding (Escobar et al., 2016; Helms et al., 2014; Henselmans et al., 2022; Slater & Phillips, 2011). At the same time, bodybuilders engage in resistance training during the preparation phase adhering to low carbohydrate and ketogenic diets to induce training adaptations, weight loss, and cardiovascular health improvement (Jeukendrup, 2017; Valenzuela et al., 2021) which may impair high‐intensity exercise performance, muscle repair, and remodeling (Margolis & Pasiakos, 2023).

Accordingly, athletes may require different amounts of carbohydrates based on their physiological and training requirements to develop a carbohydrate periodization approach to changes in nutritional intake in response to certain periods of training (Jeukendrup, 2017). Therefore, it is possible to personalize evidence‐based recommendations related to nutritional manipulations to accommodate alterations in training load and different body mass/composition goals (Mota et al., 2019). Nevertheless, before implementing carbohydrate manipulation before, during, and after resistance exercise, identifying the factors related to the change in muscle glycogen following resistance training is required to open a clearer view for making a carbohydrate periodization approach among resistance‐trained athletes.

It has previously been reported that factors including carbohydrate availability, fitness status, baseline glycogen concentration, and exercise intensity have a direct relationship with muscle glycogen concentration among endurance athletes (Areta & Hopkins, 2018). However, the factors influencing muscle glycogen depletion during resistance exercise are less well understood. Therefore, this systematic review and meta‐analysis was conducted to assess the acute effects of a resistance training session on muscle glycogen concentration. Through the present meta‐analytic approach, we considered the different moderating factors that may modulate the variation of glycogen content following a resistance training session.

## 2 METHODS

This systematic review with meta‐analysis was conducted following the Preferred Reporting Items for Systematic Reviews and Meta‐Analyses (PRISMA) guidelines (Page et al., 2021). The study protocol was pre‐registered at the International Prospective Register of Systematic Reviews (PROSPERO; identification code: CRD42023424294). The 2020 PRISMA checklist can be found in Appendix S1.

### 2.1 Search strategies

A systematic search was performed on PubMed, Web of Science, and Scopus databases from inception until 28th July 2024. The following search combinations were used through the search process: (“Resistance training” OR “Resistance exercise” OR “Weight Lift” OR “weight lifting” OR “strength training” OR Powerlift OR “resistive exercise” OR “Weight training”) AND (Glycogen OR “Muscle glycogen” OR “Glycogen depletion”). Reference lists of relevant studies were also scanned to identify other eligible studies in addition to a complemlentary search via Google Scholar. No filters in terms of language, publication year, study design, or population characteristics (e.g., sex, training status, and age) were applied. The full details of the search process can be found in Appendix S2.

### 2.2 Eligibility criteria

In the present study, consideration was given to studies meeting the following PICO criteria:

1. Participants: Healthy adult subjects (≥ 18 years old).
2. Intervention: Effect of resistance exercise on muscle glycogen concentration.
3. Comparator: Pre‐exercise muscle glycogen concentration.
4. Outcome: Glycogen concentrations measured by needle biopsy. For studies reporting multiple post‐exercise biopsies, we consistently extracted the measurement taken closest to the end of exercise.

Studies were excluded if the PICO criteria were not fulfilled. In addition, studies were excluded if:

1. Muscle glycogen data are missing.
2. Dietary constituents other than water were administered during resistance training session or feeding occurred between the exercise session and the first post‐exercise biopsy.
3. Any type of exercise was performed before resistance training.
4. The record was a review or a citation report rather than an original research study.

### 2.3 Data extraction

The primary research outcome in this study was the magnitude of glycogen depletion after a resistance training session. Values were derived as mmol·kg^−1^ of dry mass (dm), or in mmol·kg^−1^ of wet mass and subsequently adjusted by a conversion factor of 4.35 to standardize to mmol·kg^−1^ dm (Areta & Hopkins, 2018). Two independent investigators (AH and FCh) completed screening studies and data extraction from each included study using a standardized template created with Microsoft Excel. From all included studies, the following information was extracted: first author, year of publication, number of participants, participant demographics (sex), mean ± standard deviation (SD) of glycogen at pre‐ and post‐session, resistance training protocol including intensity, expressed in percentage of one repetition–maximum (% 1RM), duration and number of sets, method of glycogen measurement and biopsied muscle. Differences were resolved by consensus between the two authors. The Cohen's Kappa values for the intra‐observer agreement were higher than 0.85. In instances of missing data, the corresponding author was contacted to facilitate its retrieval. In cases where data were exclusively presented graphically, an online utility tool (“WebPlot Digitizer”, https://apps.automeris.io/wpd/) was used to extract values.

### 2.4 Risk of bias assessment

The methodological quality of the studies included in the current systematic review was assessed by two researchers (AH and JR) using the Physiotherapy Evidence Database (PEDro) scale (Cashin, 2020; Maher et al., 2003), and non‐comparative studies were assessed for quality using the National Institutes of Health (NIH) Quality Assessment Tool for before‐after (Pre‐Post) study with no control group (National Heart, Lung, and Blood Institute, 2022). The quality of studies on the PEDro scale was interpreted as follows: ≥6 (high quality), 4–5 (moderate quality), and ≤3 (low quality) (Cashin, 2020). The quality of studies on the NIH scale was interpreted as follows: Poor quality: Less than 50% of the 12 criteria are met (National Heart, Lung, and Blood Institute, 2022), fair quality: 50%–75% of the 12 criteria are met, good quality: 75% or more of the 12 criteria are met. All disagreements between reviewers were resolved by discussion. The quality assessment for each study is summarized in Tables 1 and 2.

**TABLE 1:** Risk of bias assessment using NIH Quality Assessment Tool for Before–After (Pre–Post) Studies With No Control Group.

Study	Q1	Q2	Q3	Q4	Q5	Q6	Q7	Q8	Q9	Q10	Q11	Q12	Total score	Quality rating
Tesch et al. (1986)	Y	N	Y	NR	NR	Y	Y	NR	NR	Y	NA	NA	5/10 (50%)	Fair
Robergs et al. (1991)	Y	N	Y	NR	NR	Y	Y	NR	NR	Y	NA	NA	5/10 (50%)	Fair
Hokken et al. (2021)	Y	N	Y	NR	NR	Y	Y	Y	NR	Y	NA	NA	5/10 (50%)	Fair
Harber et al. (2008)	Y	N	Y	NR	NR	Y	Y	NR	NR	Y	NA	NA	5/10 (50%)	Fair

*Note*: Q1: Was the study question or objective clearly stated?, Q2: Were eligibility/selection criteria for the study population prespecified and clearly described?, Q3: Were the participants in the study representative of those who would be eligible for the test/service/intervention in the general or clinical population of interest?, Q4: Were all eligible participants that met the prespecified entry criteria enrolled?, Q5: Was the sample size sufficiently large to provide confidence in the findings?, Q6: Was the test/service/intervention clearly described and delivered consistently across the study population?, Q7: Were the outcome measures prespecified, clearly defined, valid, reliable, and assessed consistently across all study participants?, Q8: Were the people assessing the outcomes blinded to the participants' exposures/interventions?, Q9: Was the loss to follow‐up after baseline 20% or less? Were those lost to follow‐up accounted for in the analysis?, Q10: Did the statistical methods examine changes in outcome measures from before to after the intervention? Were statistical tests done that provided *p* values for the pre‐to‐post changes?, Q11: Were outcome measures of interest taken multiple times before the intervention and multiple times after the intervention (i.e., did they use an interrupted time‐series design)?, Q12: If the intervention was conducted at a group level (e.g., a whole hospital, a community, etc.) did the statistical analysis take into account the use of individual‐level data to determine effects at the group level? NA: not applicable; NR: not reported; N: no; Y: yes. Quality Rating: Poor <50%, Fair 50%–75%, Good ≥75%.

**TABLE 2:** Risk of bias assessment using the PEDro scale for controlled studies.

Authors and Year	Criteria 1	Criteria 2	Criteria 3	Criteria 4	Criteria 5	Criteria 6	Criteria 7	Criteria 8	Criteria 9	Criteria 10	Criteria 11	Total
Coffey et al. (2009)	0	1	1	1	0	0	0	1	0	1	1	6
Pascoe et al. (1993)	0	1	1	1	0	0	0	1	0	1	1	6
Nieman et al. (2004)	1	1	0	1	1	0	0	1	0	1	1	7
Apró et al. (2015)	1	1	1	1	0	0	0	1	0	1	1	7
Moberg et al. (2016)	1	1	1	1	1	1	0	1	0	1	1	9
Fyfe et al. (2016)	1	1	1	1	0	0	0	1	0	1	1	7
Donges et al. (2014)	1	1	1	1	0	0	0	1	0	1	1	7
Breen et al. (2011)	1	1	0	1	0	0	0	1	0	1	1	6
Lundberg et al. (2012)	0	1	0	1	0	0	0	1	0	1	1	5
Lundberg et al. (2014)	0	1	0	1	0	0	0	1	0	1	1	5
Camera et al. (2010)	0	1	0	1	0	0	0	1	0	1	1	5
Camera et al. (2012)	0	1	0	1	0	0	0	1	0	1	1	5
Churchley et al. (2007)	0	0	0	1	0	0	0	1	0	1	1	3
Roy and Tarnopolsky (1998)	1	1	1	1	1	?	1	1	0	1	1	9
Carrithers et al. (2007)	1	1	1	1	0	0	0	1	0	1	1	7
Lee et al. (2023)	1	1	0	1	0	0	0	1	0	1	1	6

*Note*: 1 = eligibility criteria were specified, 2 = subjects were randomly allocated to groups, 3 = allocation was concealed, 4 = groups were similar at baseline, 5 = all subjects were blinded, 6 = therapist who administered therapy/training were blinded, 7 = all assessors who measured key outcomes were blinded, 8 = measurement of key outcomes were obtained from more than 85% of the subjects, 9 = subjects for whom outcome measures were available received the treatment or control condition as allocated or, otherwise for at least one key outcome was analyzed by “intention to treat”, 10 = results of between‐group statistical comparisons were reported for at least on key outcome, 11 = study provides both point measures and measures of variability, score: 0 = no, 1 = yes.

### 2.5 Statistical analysis

The mean difference “MD” was calculated from primary studies using the means and standard deviations from pre‐ and post‐ resistance training sessions, the sample size and the raw correlation coefficient (*r*). The MD was used as a summary statistic since glycogen depletion was measured in the same way (biopsy) in all studies (Higgins et al., 2019). For within pre‐post correlations (*r*), a 0.59 coefficient was determined from the Moberg et al. (2016) study (where authors shared their raw data) and used for analysis. Moreover, a sensitivity analysis was undertaken, trying other values (i.e., 0.3 and 0.8), to determine whether the overall result of the analysis is robust to the use of imputed correlation coefficients as recommended in the Cochrane Handbook guidelines (Higgins et al., 2019).

Because independence of effect size is crucial to avoid overlapping and provide unbiased effect size estimations (Cheung, 2014; Gucciardi et al., 2021), a multilevel meta‐analytic model was performed to handle dependent effect sizes within multi‐arms studies (Assink & Wibbelink, 2016). The multilevel, random‐effects model for meta‐analysis was performed using the “metaphor” R package (rma.mv function) (Viechtbauer, 2010) to generate an overall MD and 95% confidence interval (95% CI). Furthermore, the 95% prediction interval (PI) was calculated since it serves to find out the expected range of effect size in outlook studies (Inthout et al., 2016). Model parameters were calculated using the restricted maximum likelihood estimation method. The Q statistic and *I* ^2^ were used as indicators of heterogeneity across studies (Higgins et al., 2003). Heterogeneity was considered significant if the Q statistic reached a significance level of *p* < 0.05 (Hedges & Olkin, 2014). The *I* ^2^ value was interpreted as trivial (0%–40%), moderate (30%–60%), substantial (50%–90%), and considerable (75%–100%) (Higgins et al., 2019).

### 2.6 Moderator analysis

Moderator analysis included dichotomous and continuous variables. For dichotomous variable [i.e., training status (trained vs. untrained), intensity (fixed vs. varied)] exploratory subgroup comparisons of moderator variables were performed. For continuous variables (mean age, session duration, number of sets, rest interval between sets, exercise intensity), meta‐regression analyses were conducted. Moderator analysis was performed when there were sufficient observations per subgroup (i.e., at least 3 observations).

### 2.7 Publication bias and outlier detection

The risk of publication bias was visualized through contour‐enhanced funnel plots. Moreover, the multilevel extension of the Egger's test was used to detect publication bias with *p* < 0.1 considered as a significant threshold (Egger et al., 1997). The variance of effects was converted into standard error which was used as a predictor (Fernández‐Castilla et al., 2021). Outlier and influential case diagnostics were performed by calculating Cook's distance and standardized residuals, respectively (Cook, 1977; Viechtbauer & Cheung, 2010). Cases were considered outliers when their Cook's distance values were greater than three times their respective mean, and with a standardized residual value greater than 3, in absolute values (Gucciardi et al., 2021). In the presence of outliers, the overall effect was recalculated to assess the robustness of the fitted model.

## 3 RESULTS

### 3.1 Search result

**FIGURE 1:**
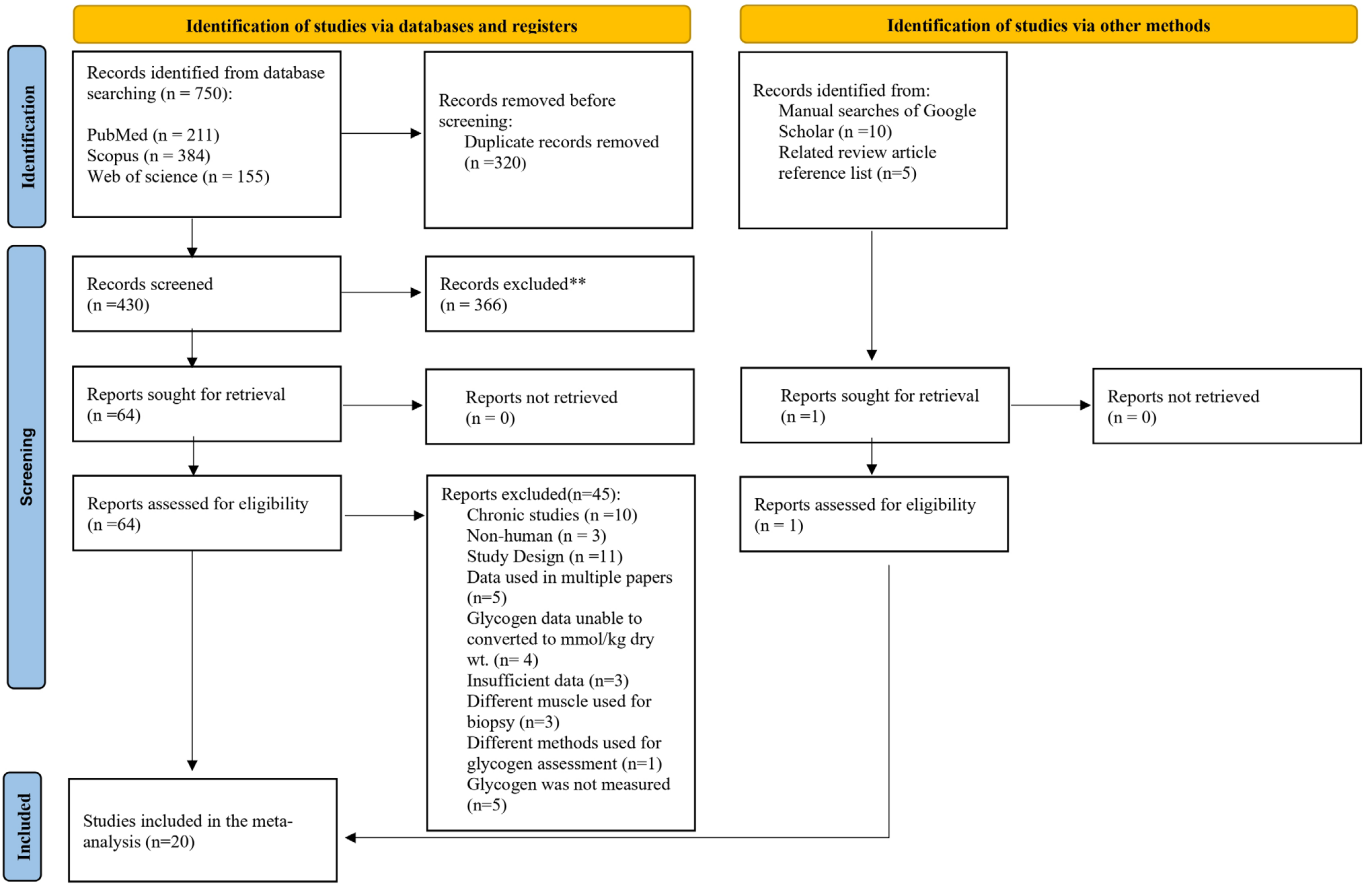
Literature search flow chart. *n*, number of studies.

The initial search yielded 750 records, of which 430 were screened by title and abstract after duplicates were removed. Title and abstract screening yielded 64 potential inclusions that were screened by full text, and 19 of these studies met the full inclusion criteria. After an additional search on Google Scholar and related review article references, one published study met the inclusion criteria, resulting in 20 studies included in this meta‐analysis. A summary of the search process is shown in Figure 1.

### 3.2 Study characteristics

The 20 studies included 180 participants (168 males, 12 females). The sample size within studies ranged from 6 (Breen et al., 2011) to 15 (Nieman et al., 2004) participants. The sample mean age was 25.6 ± 6.2 years ranging from 19.6 (Roy & Tarnopolsky, 1998) to 53.3 years. Regarding sex distribution, one study used exclusively female participants (Harber et al., 2008), one used a mixed cohort (Carrithers et al., 2007), and the remaining studies used exclusively male participants. For training status, participants were described as trained in 14 studies, and as untrained in the other ones.

In the included studies, exercise protocols comprising both free weights and machine‐based exercises were employed. The average session duration was 33.0 ± 27.0 min, with a range extending from 7 min (Lundberg et al., 2014) to 120 min (Nieman et al., 2004). Additionally, there was a diversity of loading schemes and a range of total sets completed per session. The number of sets per session was 8.7 ± 4.0 ranging from 3 (Robergs et al., 1991) to 20 sets (Tesch et al., 1998). The average rest interval between sets was 127.5 ± 44.4 s varying from 30 s (Nieman et al., 2004) to 180 s (Apró et al., 2015; Camera et al., 2010; Fyfe et al., 2016; Moberg et al., 2016). Regarding training intensities, the average intensity of the performed exercises was 72.3 ± 13.4% of 1RM, varying from 35% (Robergs et al., 1991) to 85% (Lundberg et al., 2012; Moberg et al., 2016) of 1RM. Prior to exercise, the average muscle glycogen concentration among participants was measured at 528.3 ± 261.1 mmol·kg^−1^ of dry mass, with values ranging from 276.1 (Lee et al., 2023) to 1396 mmol·kg^−1^ (Breen et al., 2011). Following exercise, the average muscle glycogen concentration decreased to 404.4 ± 204.7 mmol·kg^−1^ of dry mass, with a range from 220.3 (Roy & Tarnopolsky, 1998) to 1065 mmol·kg^−1^ (Breen et al., 2011). A comprehensive description of participant characteristics and resistance exercise protocol can be found in Table 3.

**TABLE 3:** Resistance training protocol characteristics of the studies included in the meta‐analysis.

Study	Sex (M/F)	Age (years)	Body mass (kg)	Training status	Resistance training protocol	Duration (mins)
Tesch et al. (1986)	9 M	23 ± 2	90 ± 9	Trained	5 sets each of front squat, backsquat, leg press, and knee extension to failure, 60 s rest between sets	30
Robergs et al. (1991)	8 M	23.2 ± 1.1	78.9 ± 8.4	Trained	6 × 6 leg‐extension machine at 70% 1RM, 120 s rest between sets	~15
Pascoe et al. (1993)	8 M	29 ± 1.9	80.3 ± 4.3	Untrained	Sets of 6 rep leg‐extension at 70% 1RM to fatigue, 30 s rest between sets	~8
Roy and Tarnopolsky (1998)	10 M	19.6 ± 0.69	86.8 ± 9.8	Trained	Whole‐body resistance exercise: 9 exercises/3 sets 80% 1RM	Unclear
Nieman et al. (2004)	15 M	21.3 ± 2.73	83.1 ± 14.2	Trained	Whole‐body resistance exercise: 10 exercises. 4 × 10 the first set at 40% of 1‐RM and the subsequent sets at 60% 1‐RM, 120 s rest between sets	120
Churchley et al. (2007)	7 M	30 ± 6.7	94.4 ± 14.2	Trained	8 × 5 at 80% 1 RM leg press, 180 s rest between sets	~64
Carrithers et al. (2007)	6 M, 6 F	26 ± 1	68.7 ± 2.6	Untrained	4 sets of 10 repetitions of leg press and leg extension at 80% 1RM, 120 s rest between sets, last set of each to failure	~30
Harber et al. (2008)	6 F	29 ± 7.3	76 ± 22.0	Untrained	6 × 10 of bilateral knee extensions at ~70% concentric 1 RM. 120 s rest between sets	~15
Coffey et al. (2009)	8 M	22.9 ± 6.3	73.2 ± 4.5	Trained	8 × 5 leg extension, 80% 1RM, 180 s rest between sets	~30
Camera et al. (2010)	8 M	28.4 ± 4.5	81.8 ± 15.8	Untrained	8 × 5 repetitions at approximately 80% 1RM leg extension machine, 180 s rest between sets	~30
Breen et al. (2011)	12 M	20 ± 3	79.7 ± 14.5	Untrained	8 × 10 repetitions of leg press and leg extension at 75% 1RM, 120 s rest between sets	45
Camera et al. (2012)	8 M	22.5 ± 4.4	78.2 ± 4.7	Trained	8 × 5 at 80% of 1RM leg press, 180 s rest between sets	~30
Lundberg et al. (2012)	9 M	23 ± 2	75 ± 6	Trained	2 × 7 maximal knee extension, 2 × 7 maximal leg press 90 s rest between sets	~7
Lundberg et al. (2014)	10 M	26 ± 5	77 ± 9	Trained	4 × 7maximal knee extension, 120 s rest between sets	~10
Donges et al. (2014)	8 M	53.3 ± 5.1	90.2 ± 8.7	Untrained	8 × 8 leg extension exercise at 70% of 1RM, 150 s rest between sets	24
Apró et al. (2015)	8 M	26 ± 5.6	85 ± 5.6	Trained	4 sets of 8–10 repetitions at 80% 1RM, 4 sets of 10–12 repetitions at 70% 1RM, 2 sets to volitional fatigue at 60% 1RM leg press, 180 s rest between sets	60
Fyfe et al. (2016)	8 M	27 ± 4	83.7 ± 13.7	Trained	8 × 5 unilateral leg press repetitions on each leg at 80% of the 1RM 1RM by 60 s rest between sets	~20
Moberg et al. (2016)	8 M	27 ± 5.6	84 ± 8.4	Trained	10 sets of heavy seated in the leg press to fatigue, starting at 85% of their 1RM and gradually reducing the load by 3 min of rest	50
Hokken et al. (2021)	10 M	24 ± 4	95 ± 8	Trained	4 × 5 repetitions at 75% of 1RM back squats and deadlifts and 4 × 12 at 65% of 1RM rear foot elevated split squats.	70–90
Lee et al. (2023)	8 M	27 ± 5	73.7 ± 9.5	Trained	6 × 10 leg press repetitions at 70% 1‐RM, separated by 2‐min rest periods on a plate‐loaded 45° incline leg press	20–25

*Note*: Values are expressed as means ± standard deviations.

Abbreviations: 1RM, one repetition maximum; *M*, male; *F*, female; *S*, seconds.

### 3.3 Meta‐analysis results

**FIGURE 2:**
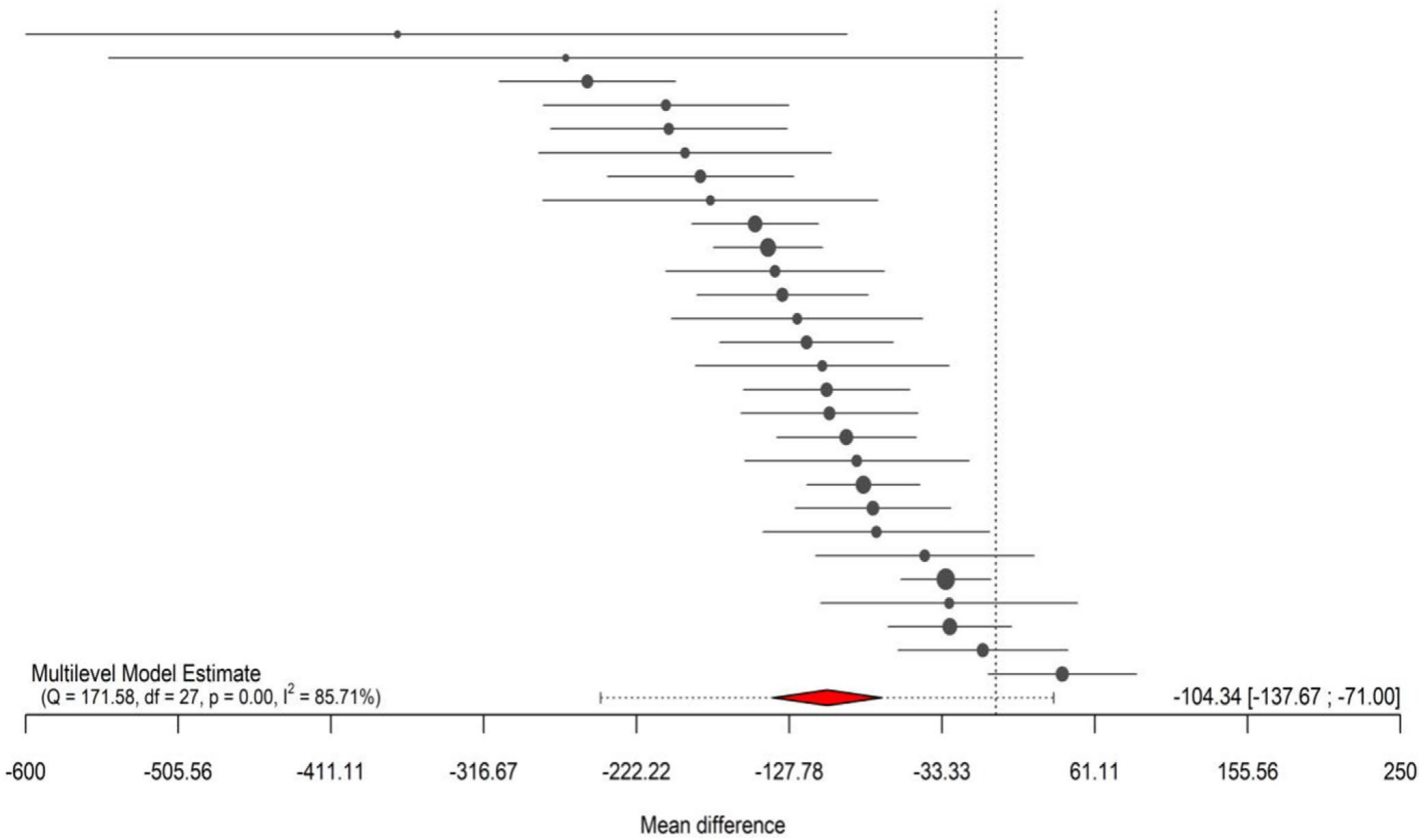
Forest Plot of multilevel meta‐analysis assessing the influence of resistance exercise on muscle glycogen depletion. The circle represents the pooled estimate with 95% confidence interval (95% CI). The red diamond represents the overall effect estimate with 95% prediction interval (95% PI).

**FIGURE 3:**
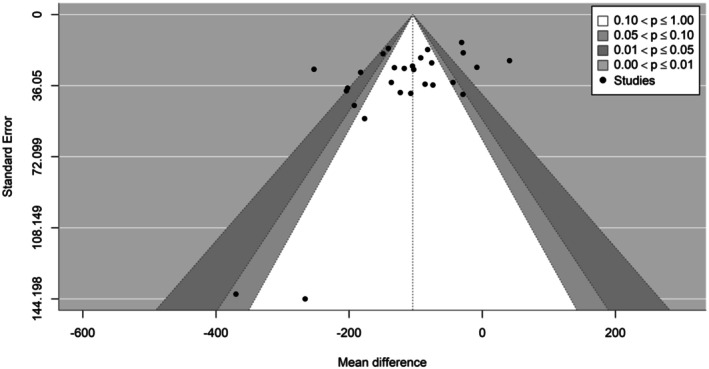
Contour‐enhanced funnel plot of studies included in the meta‐analysis.

The fitted model (28 effect sizes across 20 clusters) showed significantly greater glycogen depletion (MD = −104.3; 95% CI: −137.6 to −71.0; *p* < 0.001; PI: −244.4 to 35.7), with high heterogeneity (Q_27_ = 171.5; *p* < 0.001; *I* ^2^ = 85.7%) (Figure 2). The visual inspection of the funnel plot indicated a seemingly non‐symmetrical distribution pattern of the effects (Figure 3). The Egger test identified a possible risk of publication bias (*β* = −44.7; CI: −98.8 to 9.3; *z* = −2.6; *p* = 0.009) (Tables 1 and 2).

**FIGURE 4:**
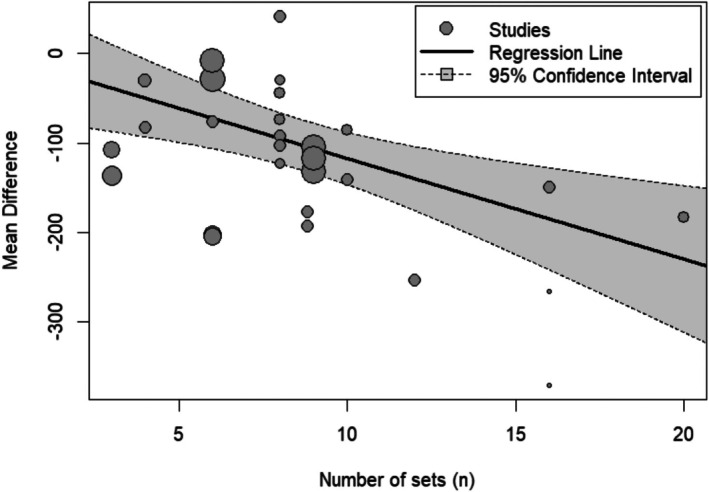
Multilevel meta‐regression analysis examining the relationship between the number of sets and mean glycogen depletion across studies. The regression line indicates a negative association between the number of sets and glycogen levels, with larger mean differences observed as the number of sets increases. The shaded area represents the 95% confidence interval, while each data point reflects an individual study, with the size of the points corresponding to the study's weight in the analysis.

Meta‐regression showed the number of sets moderates the effect of resistance training on glycogen depletion, with mean difference increasing with the number of sets (Estimate = −11.2; 95% CI: −18.0 to −4.3; *p* = 0.001). The analysis further revealed high heterogeneity (*I* ^2^ = 81.2%) (Figure 4).

**FIGURE 5:**
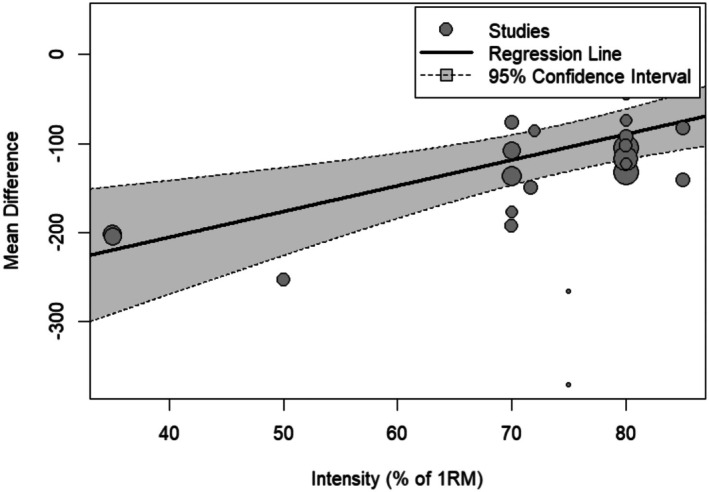
Multilevel meta‐regression analysis examining the relationship between exercise intensity and mean glycogen depletion across studies. The regression line indicates a positive association between the intensity and glycogen depletion levels, with smaller mean differences observed as the intensity increases. The shaded area represents the 95% confidence interval, while each data point reflects an individual study, with the size of the points corresponding to the study's weight in the analysis. 1RM, one repetition maximum.

Moreover, glycogen depletion decreased with exercise intensity (Estimate = 2.8; 95% CI: 1.2–4.5; *p* = 0.0006). The analysis further revealed high heterogeneity (*I* ^2^ = 77.0%) (Figure 5).

**FIGURE 6:**
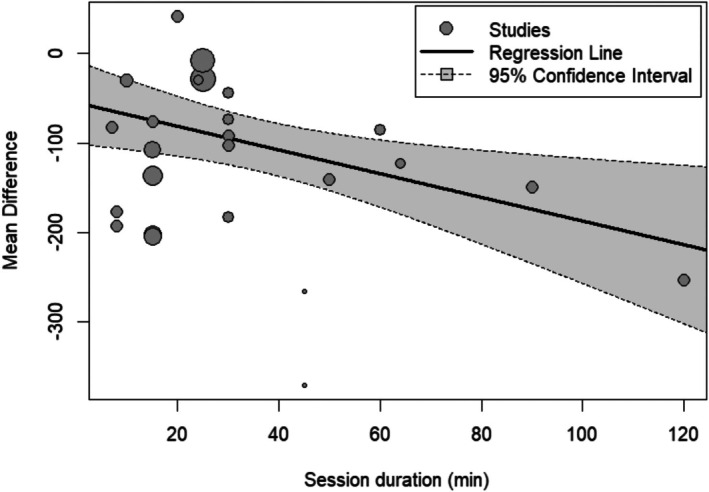
Multilevel meta‐regression analysis examining the relationship between the session duration and mean glycogen depletion across studies. The regression line indicates a negative association between the session duration and glycogen depletion levels, with larger mean differences observed as the session duration increases. The shaded area represents the 95% confidence interval, while each data point reflects an individual study, with the size of the points corresponding to the study's weight in the analysis.

Glycogen depletion also increased with session duration (Estimate = −1.3; 95% CI: −2.3 to −0.3; *p* = 0.009). The analysis further revealed high heterogeneity (*I* ^2^ = 81.3%) (Figure 6).

However, there were no effects of the between sets recovery (MD = 0.70; 95% CI: −0.06 to 1.47; *p* = 0.07), or age (MD = 4.03; 95% CI: −0.82 to 8.89; *p* = 0.10).

Subgroup analysis showed a significant difference between intensity subgroups (*p* < 0.001) with greater glycogen depletion recorded under the varied intensity (MD = −162.9; 95% CI: −216.6 to −109.2; *p* < 0.001) than the fixed one (MD = −82.5; 95% CI: −114.4 to −50.7; *p* < 0.001). Analysis further showed high heterogeneity (*I* ^2^ = 80.2%). Regarding training status, there was a significant difference between subgroups (*p* < 0.001) with greater glycogen depletion recorded within untrained subjects (MD = −113.0; 95% CI: −176.4 to −49.8; *p* = 0.0005) than within trained ones (MD = −101.3; 95% CI: −139.7 to −63.0; *p* < 0.0001). Analysis further showed high heterogeneity (*I* ^2^ = 86.4%).

Outlier and influential case diagnostics showed no existing outlier residuals. However, Cook's outliers' analysis revealed 2 outliers (Fyfe et al., 2016; Nieman et al., 2004) that their exclusion did not affect the fitted model (MD = −103.0; 95% CI: −129.8 to −76.2; *p* < 0.001; PI = −203.8 to −2.2) but reduced heterogeneity (*I* ^2^ = 74.9).

### 3.4 Sensitivity analysis

As presented in Table 4, the overall result of the analysis was relatively insensitive to the imputed correlation coefficient indicating the robustness of the fitted model.

**TABLE 4:** Results of the sensitivity analysis.

	*r* = 0.30				*r* = 0.80			
	MD [95% CI]	[PI]	*p* Value	*I* ^2^	MD [95%CI]	[PI]	*p* Value	*I* ^2^
Overall	−103.3 [−136.3; −70.2]	[−235.6; 28.9]	<0.001	76.6%	−105.4 [−139.2; −71.6]	[−252.9; 42.0]	<0.001	92.4%

Abbreviations: 95% CI, 95% confidence interval; MD, mean difference; PI, prediction interval.

## 4 DISCUSSION

This systematic review with meta‐analysis is the first to synthesize evidence quantifying skeletal muscle glycogen concentration following a resistance training session. The fitted meta‐analytic model indicates that muscle glycogen content decreased significantly after resistance training. Meta‐regression analysis revealed that glycogen depletion increased with the number of sets and session duration while decreasing as exercise intensity increased. Subgroup analysis showed greater glycogen depletion under varied intensity than a fixed one, and within untrained subjects compared with trained ones.

### 4.1 Influence of resistance training session on muscle glycogen utilization

The present meta‐analysis indicates that one session of resistance training elicited a significant reduction in muscle glycogen concentration by ~21%. While a notable decline in muscle glycogen has been documented subsequent to a resistance training session, the extent to which this depletion may impair performance remains uncertain. It has been suggested that when muscle glycogen falls below a critical level of muscle glycogen (typically ~280–300 mmol. kg^−1^dw) the release of calcium from the sarcoplasmic reticulum is impaired, impacting muscle contraction and potentially leading to fatigue (Ørtenblad et al., 2011). However, after a typical resistance training session muscle glycogen is still likely to be above that critical level. For example, Essén et al. (Essen‐Gustavsson & Tesch, 1990) found vastus lateralis glycogen decreased from 690 to 495 mmol kg^−1^ dm in nine bodybuilders following five sets each of front squats, back squats, leg presses, and leg extensions to failure at ~12 rep max (RM). Thus, glycogen depletion from typical resistance training workouts is relatively modest and does not deplete enough glycogen to compromise performance. However, most studies measured whole‐muscle glycogen levels, but the average value may not reflect the intramyofibrillar glycogen stores, which appear to have the greatest impact on muscle function. Notably, following resistance training, glycogen stores undergo nonhomogeneous depletion across distinct subcellular compartments and fiber types (Hokken et al., 2021). In type I fiber only intermyofibrillar glycogen decreased significantly after resistance exercise (−33%), while in type II fibers significant reductions were observed in all three distinct compartments (Hokken et al., 2021). Specifically, 48% of the type II fibers demonstrated near depleted levels of intramyofibrillar glycogen after the exercise session (Hokken et al., 2021). In the present meta‐analysis the vastus lateralis was the targeted muscle. The vastus lateralis is a large, pennate muscle, typically the largest of the quadriceps femoris muscles, primarily active for knee extension (Oatis, 2009). Given its size and primary function, the vastus lateralis is heavily recruited during resistance training exercises such as squats, leg presses, and other knee‐extension exercises (Martín‐Fuentes et al., 2020). This makes it a critical muscle to study when assessing glycogen depletion due to resistance training. However, Horwath et al. (2021) reported large coefficients of variation between vastus lateralis biopsy samples in type I and type II fibers (13% and 14%, respectively), with results deviating by as much as 42%–48% within subjects. This raises skepticism regarding the confidence that can be placed on the current data when attempting to draw evidence‐based inferences.

### 4.2 Moderating factors of glycogen depletion

#### 4.2.1 Effect of training duration and intensity

The current meta‐regression analyses showed that glycogen depletion increases with the number of sets and session duration. While the long‐term impact of training volume on muscular adaptations like hypertrophy and strength is widely documented (Schoenfeld et al., 2018), our current findings highlight the acute consequences of long duration training on muscle glycogen levels. Low glycogen levels impair the muscle's ability to regenerate adenosine triphosphate (ATP), which is critical for sustaining muscle contractions (Knuiman et al., 2015). When glycogen stores are depleted, the rate of ATP production diminishes, leading to reduced force output and increased fatigue (Ørtenblad et al., 2013). Specifically, it has been demonstrated that a reduction of muscle glycogen negatively affects isokinetic torque. This relationship highlights the importance of maintaining adequate glycogen levels for optimal performance (Jacobs et al., 1981).

Regarding exercise intensity, the meta‐regression analysis revealed glycogen utilization decreases with increased exercise intensity. This might be related to the lower volume of work that can be performed with heavier loads (Schoenfeld et al., 2021). It has also been observed that the rate of carbohydrate utilization is reduced with repeated bouts of high‐intensity exercise (Bogdanis et al., 1996), possibly related to muscle acidosis, the increased reliance on aerobic energy metabolism, and/or a result of the decrease in work capacity and; therefore, energy requirements caused by fatigue development (Spriet, 1992). In addition, the subgroup analysis revealed significantly greater glycogen depletion under varied intensity conditions compared to fixed intensity. A key factor contributing to this difference appears to be the substantially higher training volume in studies utilizing varied intensities. Specifically, the average number of sets was approximately 13.5 in the varied load studies, compared to around 7.5 sets in those with fixed loads. This greater training volume likely increased the overall energy demand, resulting in more pronounced glycogen depletion.

#### 4.2.2 Effects of training status

The subgroup analysis showed higher glycogen depletion within untrained subjects than within trained ones, indicating a potential impact of training status on metabolic responses during resistance exercise. This finding aligns with previous research suggesting that although the relative use of energy sources during exercise is mainly determined by the intensity and the duration, an individual's training status also contributes to this phenomenon (Hearris et al., 2018).

While resistance exercise is primarily known for promoting strength and hypertrophy through mechanical stimulation, its aerobic effects have also been investigated (Hong et al., 2024; Ozaki et al., 2013). It has been recently shown that squat exercise can be considered an exercise training modality with substantial involvement of aerobic energy metabolism (Hong et al., 2024). Interestingly, research suggests that resistance exercise can trigger myocellular signaling for mitochondrial biogenesis, though to a lesser extent than endurance training, depending on factors like training status and exercise protocols (Groennebaek & Vissing, 2017). One possible mechanism for the reduced glycogen utilization in trained individuals may be the enhancement of oxidative phosphorylation, resulting in a decreased reliance on muscle glycogen. However, this requires further exploration in future long‐term training studies. In addition, decreased antagonist muscle co‐activation as a result of resistance training may contribute to improved movement efficiency in trained individuals (Kuruganti et al., 2006). Therefore, lower glycogen utilization in trained athletes can be attributed not only to enhanced oxidative capacity but also to more efficient neuromuscular coordination. This improved coordination may reduce unnecessary energy expenditure during movements, allowing for a more effective utilization of energy reserves (Shahtout et al., 2020).

### 4.3 Review strength and limitations

This is the first systematic review with meta‐analysis to explore the acute effect of a resistance training session on muscle glycogen utilization. While the study was conducted respecting the Cochrane handbook for systematic reviews of interventions and the updated PRISMA guidelines, there were some limitations that should be acknowledged. Firstly, in the present study, due to the limited number of studies and variability between them, a meta‐analysis on glycogen utilization from distinct storage compartments following resistance training could not be performed. While glycogen depletion in the intramyofibrillar storage can likely begin impairing performance at relatively modest levels of overall muscle glycogen depletion. Secondly, we were unable to perform other meta‐regression or subgroup analyses for several other moderating factors such as the number of repetitions, time of day, and sex due to the limited data available. Furthermore, these data represent whole‐muscle glycogen content and do not delineate both fiber type and subcellular heterogeneity in utilization as outlined above.

### 4.4 Studies limitations and perspectives

The majority of studies focused exclusively on male participants, with only one study including female participants (Harber et al., 2008). This sex disparity limits the generalizability of our findings and may introduce a potential source of bias, as females have a well‐established reduction in carbohydrate use during endurance exercise (Rothschild et al., 2022; Tarnopolsky, 2000). Future research should strive for more balanced gender representation to make findings more generalizable. Moreover, focusing solely on the vastus lateralis limits the generalizability of these findings, as glycogen utilization patterns may differ in other muscle groups. In addition, given that the studies have explored the impact of resistance training on glycogen depletion in the vastus lateralis, it is imperative to note that different storage depots within muscles may exert distinct effects on muscle function and fatigue (Ørtenblad et al., 2013). Therefore, it is strongly advised that future research endeavors prioritize the investigation of glycogen utilization across different storage compartments following resistance training, such as the biceps (MacDougall et al., 1999b) and soleus (Trappe et al., 2004). This approach will offer valuable insights into the nuanced mechanisms underlying muscle metabolism and provide a more comprehensive understanding of the physiological adaptations induced by resistance exercise. Importantly, additional methodological limitations should be considered including the lack of randomized order of exercises, dietary control, and follow‐up procedures.

## 5 CONCLUSION

The present systematic review with meta‐analysis showed that one session of resistance training significantly increases glycogen depletion in the vastus lateralis muscle. The glycogen depletion increased with more sets and extended session duration while decreasing with exercise intensity. Moreover, training status modulates glycogen utilization with greater depletion recorded within untrained subjects than trained ones. In addition, the variation in exercise intensity may amplify the level of depletion compared to using a constant intensity. While typical training does not lower glycogen to levels that impair performance, the average measurements may not reflect critical intramyofibrillar stores. However, the documented findings can't be generalized and future research should address gender disparities and focus on different glycogen storage compartments for a more comprehensive understanding.

## AUTHOR CONTRIBUTIONS

AH and AJ conceived and designed the review. AH performed database searches. FC and AH participated in the screening process. AH, JR, and FC participated in data extraction and risk of bias assessment. SD and AH performed the meta‐analyses and meta‐regressions. SD was the primary author of the abstract and results section. AN and AH wrote the first draft of the manuscript. All authors critically revised the manuscript and approved the final version of the manuscript.

## FUNDING INFORMATION

No sources of funding were used to assist in the preparation of this article.

## CONFLICT OF INTEREST STATEMENT

No conflicts of interest to declare.

## ETHICS STATEMENT

Not applicable.

## Supporting information


Appendix S1.

Appendix S2.

## Data Availability

The datasets and associated code for the analyses performed are available on the Open Science Framework (https://osf.io/s7z6t).
