# Supplementary material for: Acute effects of resistance exercise on skeletal muscle glycogen depletion: A systematic review and meta‐analysis
Source: Physiol Rep. 2025 Dec 19;13(24):e70683. doi: 10.14814/phy2.70683 (PMC12717450; doi:10.14814/phy2.70683)
Supplement: Supplementary file 2 — Appendix S2. [file PHY2-13-e70683-s002.docx]

**Title**

Acute effects of resistance exercise on skeletal muscle glycogen depletion: a systematic review and meta-analysis

**Journal**

Physiological reports

**Authors**

Amin Hamidvand^1^, Slaheddine Delleli^2,3^, Jeffrey A. Rothschild^4,5^, Farzaneh Chenaghchi^6^, Afshar Jafari^1^, Alireza Naderi^7^

**Affiliations**

^1^Department of Biological Sciences in Sport, Faculty of Sport Sciences and Health, Shahid Beheshti University, Tehran, Iran

^2^High Institute of Sport and Physical Education, University of Sfax, Sfax 3038, Tunisia

^3^Physical Activity, Sport, and Health, UR18JS01, National Observatory of Sport, Tunis 1003, Tunisia

^4^Sports Performance Research Institute New Zealand, Auckland University of Technology, Auckland, New Zealand

^5^High Performance Sport New Zealand, Auckland, New Zealand

^6^Department of Exercise Physiology, Faculty of Sport Sciences, University of Guilan, Rasht Iran.

^7^Department of Sport Physiology, Faculty of Human Sciences, Borujerd Branch, Islamic Azad University, Borujerd, Iran; [naderi_a@yahoo.com](mailto:naderi_a@yahoo.com)

**Corresponding author**

Alireza Naderi

Department of Sport Physiology, Faculty of Human Sciences, Borujerd Branch, Islamic Azad University, Borujerd, Iran Email: [naderi_a@yahoo.com](mailto:naderi_a@yahoo.com)

**Search Strings**

Scopus:

( TITLE-ABS-KEY ( "Resistance training" OR "Resistance exercise" OR "Weight Lift" OR "strength training" OR powerlift OR "resistive exercise" OR "weight lifting" OR "Weight training" ) ) AND ( TITLE-ABS-KEY ( glycogen OR "Muscle glycogen" OR "Glycogen depletion" ) )

Web of science

AB=("Resistance training" OR "Resistance exercise" OR "Weight Lift" OR "strength training" OR Powerlift OR "resistive exercise" OR "weight lifting" OR "Weight training")

Pubmed:

("Resistance training"[All Fields] OR "Resistance exercise"[All Fields] OR "Weight Lift"[All Fields] OR "strength training"[All Fields] OR (("power, psychological"[MeSH Terms] OR ("power"[All Fields] AND "psychological"[All Fields]) OR "psychological power"[All Fields] OR "power"[All Fields] OR "powered"[All Fields] OR "powers"[All Fields] OR "powering"[All Fields]) AND ("lifting"[MeSH Terms] OR "lifting"[All Fields] OR "lift"[All Fields])) OR "resistive exercise"[All Fields] OR "weight lifting"[All Fields] OR "Weight training"[All Fields]) AND ("glycogen"[MeSH Terms] OR "glycogen"[All Fields] OR "glycogenic"[All Fields] OR "glycogens"[All Fields] OR "Muscle glycogen"[All Fields] OR "Glycogen depletion"[All Fields])
